# A Semimetal-Like Molybdenum Carbide Quantum Dots Photoacoustic Imaging and Photothermal Agent with High Photothermal Conversion Efficiency

**DOI:** 10.3390/ma11091776

**Published:** 2018-09-19

**Authors:** Wenhao Dai, Haifeng Dong, Xueji Zhang

**Affiliations:** 1 Research Center for Biomedical and Health Science, Anhui Science and Technology University, Fengyang 233100, China; daiwenhao734028@126.com; 2 Research Center for Bioengineering and Sensing Technology, School of Chemistry and Bioengineering, University of Science & Technology Beijing, Beijing 100083, China

**Keywords:** molybdenum carbide, quantum dots, theranostic nanomedicines, dual-modal imaging, photothermal therapy

## Abstract

Theranostic platforms integrating imaging diagnostic and therapeutic interventions into a single nanoplatform have attracted considerable attention for cancer-individualized therapies. However, their uncertain stability, complex pharmacokinetics, and intrinsic toxicology of multiple components hinder their practical application in clinical research. In this paper, stable and high-concentration molybdenum carbide quantum dots (Mo_2_C QDs) with a diameter of approximately 6 nm and a topographic height of about 1.5 nm were synthesized using a facile sonication-assisted liquid-phase exfoliation approach. The prepared Mo_2_C QDs exhibited a strong near-infrared (NIR) absorbance with a high molar extinction coefficient of 4.424 Lg^−1^cm^−1^ at 808 nm, a high photothermal conversion efficiency of 42.9%, and showed excellent performance on photoacoustic imaging. The Mo_2_C QDs had high stability and highly biocompatibility, with low cytotoxicity. Under NIR irradiation, a remarkable in vitro and in vivo therapeutic effect was obtained. Such a stable and biocompatible all-in-one theranostic nanoagent generated by facile synthesis that combines promising imaging guidance and effective tumor ablation properties may hold great potential for theranostic nanomedicine.

## 1. Introduction

Nanotechnologies have played significant role in the development of both diagnostic and therapeutic agents and have contributed to the emergence of therapeutic examples expected to contribute to precise medicine [1,2,3,4,5,6,7]. In general, theranostic nanomedicines utilize the capacity of nanoplatforms to package imaging and treatment moieties onto them with both diagnostic and therapeutic properties [8,9,10,11,12]. The resulting nanosystems allow precise diagnosis and efficient therapeutics. However, concerns about their uncertain architecture stability, complex pharmacokinetics, and potential increased toxicology remain a formidable challenge for their clinical application [13,14,15,16,17,18,19]. Therefore, it is essential to achieve single theranostic nanoparticles with intrinsic imaging and therapeutic capabilities [20]. 

Thermal ablation cancer therapy is a simple, safe, and minimally invasive treatment technique based on partial extreme temperature, which directly treats irreversible injury and coagulation necrosis of tumor cells [21,22]. Various photothermal therapy (PTT) agents with excellent photothermal conversion efficiency and biocompatibility have been developed. In general, photothermal nanomaterials for PTT fall into two broad categories: inorganic photothermal nanomaterials and organic photothermal nanomaterials. Inorganic photothermal nanomaterials exhibit advantageous features, including high near-infrared absorption capacity, easy preparation and functionalization as well as a range of imaging capabilities. Various noble metal nanoparticles [21,23], metallic sulfide nanomaterials [24], carbon-based nanomaterials [4], and two dimensional transition metal carbides or nitrides (MXenes) [25] have all attracted significant interest.

The two-dimensional (2D) ultrathin molybdenum carbide (Mo_2_C) belongs to the new family of MXenes and has attracted considerable interest due to its intriguing properties [26,27]. Mo_2_C—where the carbon atom layer is sandwiched between two molybdenum atoms layers by covalent forces—exhibits semimetal-like properties. On the one hand, it has high melting point, hardness, wear resistance, and oxidation resistance; on the other hand, it shows excellent electronics and optoelectronics activity for energy storage and catalysis [28,29,30,31]. However, to the best of our knowledge, the biomedical applications of Mo_2_C nanomaterials have never been explored.

**Figure 1:**
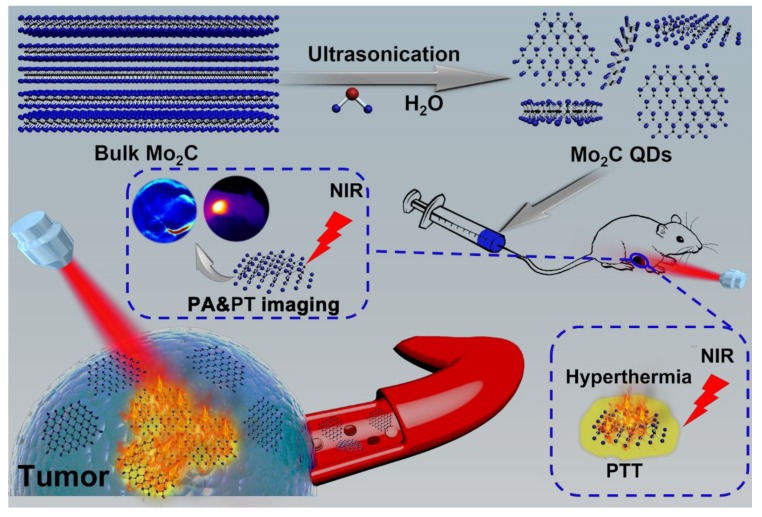
Schematic diagram of liquid exfoliation of molybdenum carbide quantum dots (Mo_2_C QDs) and in vivo photoacoustic (PA)/photothermal (PT) imaging-guided photothermal therapy (PTT) for cancer.

In this work, for the first time, water-soluble monolayered and high-concentration Mo_2_C quantum dots (QDs) were prepared through a facile liquid exfoliation method (Figure 1). The obtained ultrasmall Mo_2_C QDs exhibited a strong, broad NIR absorption with a high molar extinction coefficient and good photothermal conversion efficiency owing to the localized surface plasmon resonance (LSPR) effect. These Mo_2_C QDs were employed as nanotheranostic agents for photoacoustic (PA)/photothermal (PT) imaging-guided PTT for cancer.

## 2. Materials and Methods

### 2.1. Synthesis of Mo_2_C QDs

The Mo_2_C QDs were synthesized as previously reported, with some modification [32]. Briefly, 1 g Mo_2_C powder was dispersed in 200 mL ultrapure water and sonicated (400 W) for 20 h at room temperature. After centrifugation at 5000 rpm for 15 min to remove the flakes of Mo_2_C, the resulting supernatant was filtered through a 0.22 µm microporous membrane (Envta Technology, Beijing, China), and the Mo_2_C QDs solution was obtained.

### 2.2. In Vitro Phototoxicity Assay and Confocal Imaging

In vitro photothermal effect of Mo_2_C QDs was measured by 3-(4,5-dimethyl-2-thiazolyl)-2,5-diphenyl-2-H-tetrazolium bromide (MTT) assays on B16-F10 cells. B16-F10 cells were cultured for 12 h in a 96-well plate (WHB Scientific, Shanghai, China). 100 μg/mL Mo_2_C QDs (diluted in Opti-MEM (Gibco, Beijing, China)) or pure Opti-MEM were then added to the wells and co-cultured for 4 h. After incubation for another 8 h, the cells were exposed to an NIR laser (808 nm, 0.64 W/cm^2^, LOS-BLD-0808-2W-C/P, Hi-Tech Optoelectronics, Beijing, China) for 0–10 min and then incubated for another 24 h.

Confocal imaging was performed with an Olympus FV1200 laser scanning confocal microscope (FV1200, Olympus, Tokyo, Japan). The B16-F10 cells (5 × 10^5^) transfected with Mo_2_C QDs were cultured in a confocal dish, irradiated with an 808 nm laser (0.64 W/cm^2^, 10 min), and incubated with calcein-AM and PI solution for 30 min.

### 2.3. In Vitro and In Vivo PA Imaging

A series of Mo_2_C QDs solutions with different concentrations were loaded into agar gel cylinders with a diameter of about 1.0 cm for PA signal detection. Female BALB/c nude mice were purchased from Peking University Health Science Center (Beijing, China). The Institutional Animal Care and Use Committee of the Beijing Institute of Basic Medical Science (Beijing, China) approved all the animal experiments. Tumor-bearing nude mice were intravenously injected with Mo_2_C QDs solution, and PA signal was recorded on a multispectral optical tomography system (MSOT in Vision 128, iThera Medical, Munich, Germany). 

### 2.4. In Vivo Photothermal Therapy

When the tumor volume reached ~100 mm^3^, BALB/c tumor-bearing nude mice were divided into four groups (*n* = 5): The first group was intravenously injected with phosphate buffer saline (PBS, pH 7.4, 10 mM) as control group; the second group was intravenously injected with Mo_2_C QDs in PBS; the third group was only irradiated by NIR laser (808 nm); and the fourth group was intravenously injected with Mo_2_C QDs and then exposed to 808 nm laser. Four hours after intravenous injection, the nude mice were anesthetized and irradiated with 808 nm laser (0.64 W/cm^2^, 10 min). The tumor volume was calculated according to the formula: length × width^2^/2. Moreover, the tumor issue were sliced and stained for histological analysis.

## 3. Results and Discussions

**Figure 2:**
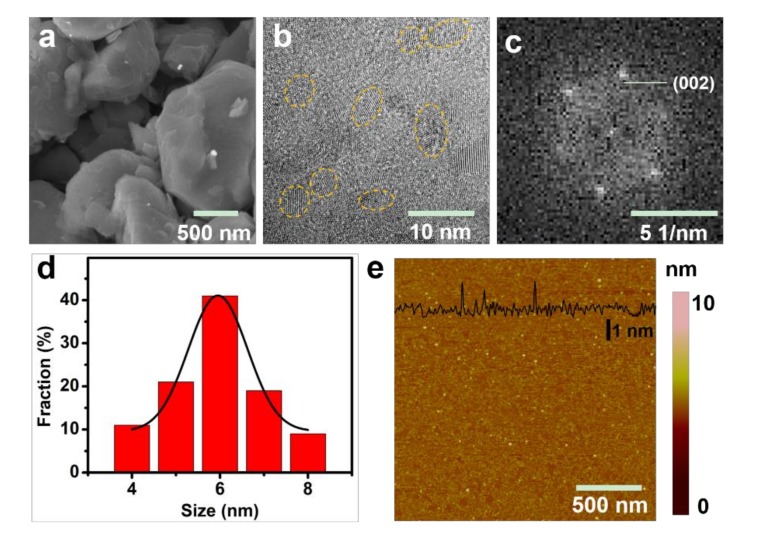
(**a**) SEM image; (**b**) TEM image of Mo_2_C QDs and (**c**) the corresponding fast Fourier transform (FFT) patterns of (**b**); (**d**) The diameter distribution analysis of Mo_2_C QDs; (**e**) Atomic force microscope (AFM, Multimode III, Bruker, Billerica, MA, USA) image of Mo_2_C QDs.

The micrometer-size Mo_2_C powder was used as precursor to synthesize the monolayered Mo_2_C QDs. As shown in Figure 2a, the Mo_2_C powder clearly displayed a multilayer flat structure and the transmission electron microscopy (TEM, FEIF20, FEI, Hillsboro, OR, USA) image of the Mo_2_C QDs indicated the monodisperse crystalline with a uniform diameter of ≈6 nm (Figure 2b,d). The fast Fourier transform (FFT, FEIF20, FEI, Hillsboro, OR, USA) pattern clearly showed the hexagonal crystalline structure of the Mo_2_C QDs (Figure 2c). As expected, the as-prepared Mo_2_C QDs possessed an average thickness between 1 and 1.5 nm (Figure 2e), corresponding to 1–2 layers of Mo_2_C QDs.

**Figure 3:**
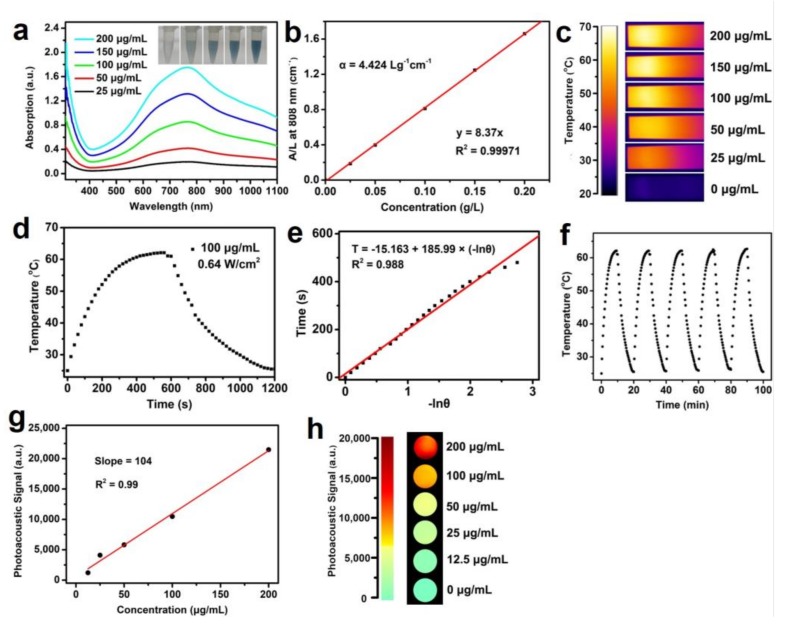
(**a**) UV-vis−NIR absorbance spectra of Mo_2_C QDs in aqueous solutions at varied concentrations. Insets are the photographs of Mo_2_C QDs with different concentrations (25, 50, 100, 150, and 200 μg/mL); (**b**) Normalized absorbance intensity for λ = 808 nm at different concentrations; (**c**) Thermal infrared images of Mo_2_C QDs in aqueous solution under 808 nm laser irradiation; (**d**) Photothermal curves of Mo_2_C QDs solutions irradiated with a 808 nm laser (0.64 W/cm^2^); (**e**) The linear time data versus −lnθ obtained from the cooling curve; (**f**) Photothermal stability of Mo_2_C QDs solution (100 μg/mL) over five NIR laser on/off cycle irradiation; (**g**) The linear correlation between photoacoustic (PA) intensities and Mo_2_C QDs concentrations in the range from 0 to 200 μg/mL; (**h**) PA imaging phantoms of Mo_2_C QDs at various concentrations.

The UV-vis−NIR absorption spectra (Figure 3a) obtained with the Mo_2_C QDs showed a strong LSPR peak in NIR region ranging from 700 to 850 nm, similar to TiS_2_ nanosheets [33] and Ti_3_C_2_ nanosheets [34]. It has been demonstrated that Mo_2_C consisting of two molybdenum atoms inserted with a layer of carbon atom exhibits semimetal-like energy band structure and NIR absorbance peak from LSPR is assigned to an indirect interband transition [33,34,35]. As shown in Figure 3b, the extinction coefficient of the as-prepared Mo_2_C QDs at 808 nm was estimated to be 4.424 Lg^−1^ cm^−1^, which was higher than carbon nanodots (0.35 Lg^−1^ cm^−1^) and superior to graphene oxide nanosheets (3.6 Lg^−1^ cm^−1^) and gold nanorods (3.9 Lg^−1^ cm^−1^) at 808 nm [36,37]. 

The efficient NIR absorption of the Mo_2_C QDs in the 700–850 nm inspired us to further investigate its potential application as promising photothermal and PA imaging agent. As shown in Figure 3c,d, as expected, the temperature of the Mo_2_C QDs solution increased by 36 °C at low concentration (100 μg/mL), indicating that the Mo_2_C QDs could efficiently convert NIR light into thermal energy. The photothermal conversion efficiency (η) of Mo_2_C QDs was calculated to be 42.9% (Figure 3e), which is remarkably higher than that of traditional gold nanorods (21%) [38], black phosphorus quantum dots (28.4%) [39], and Ti_3_C_2_ nanosheets (30.6%) [34]. The photothermal performance of the Mo_2_C QDs did not display obvious deterioration during the five heating–cooling cycles, highlighting the good photothermal stability and reproducibility of Mo_2_C QDs as a photothermal agent (Figure 3f). 

PA imaging is an emerging noninvasive imaging method that combines the advantages of high selectivity of optical tissue imaging and deep tissue penetration of ultrasound imaging [40]. We first explored the in vitro PA signals of Mo_2_C QDs at various concentrations of Mo_2_C QDs, and the results (Figure 3g,h) revealed a concentration-dependent PA signal pattern. The PA intensity showed a good linear relationship with the Mo_2_C QDs concentration in the range from 0 to 200 μg/mL.

**Figure 4:**
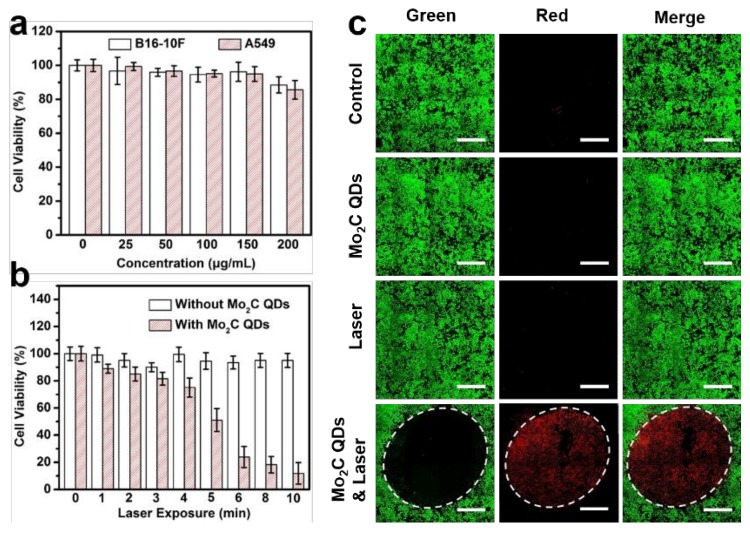
(**a**) Cell viabilities of B16-10F and A549 cells incubated with Mo_2_C QDs at different concentrations for 24 h; (**b**) Cell viabilities of B16-10F cells incubated with or without Mo_2_C QDs under an 808 nm laser (0.64 W/cm^2^) irradiation for different times; (**c**) Confocal images of calcein AM/PI-costained B16-10F cells incubated with or without Mo_2_C QDs and treated with or without an 808 nm laser (0.64 W/cm^2^) for 10 min. Scale bars: 1 mm.

In vitro cytotoxicity of the Mo_2_C QDs was evaluated by mouse melanoma B16-10F cells and human non-small cell lung cancer A549 cells. Negligible cytotoxicity was observed for both of the two cell lines when the concentration increased to 200 μg/mL, demonstrating the good biocompatibility of Mo_2_C QDs (Figure 4a). Figure 4b shows that the 808 nm laser (0.64 W/cm^2^) irradiation produced slight cytotoxicity to B16-10F cells in 10 min. By contrast, the Mo_2_C QDs-incubated B16-10F cells displayed high photocytotoxicity, and over 90% cells were killed after 10 min irradiation due to the photothermal effect of Mo_2_C QDs. The results of calcein AM (living cells, green fluorescence) and propidium iodide (PI; dead cells, red fluorescence) co-staining assay was consistent with of the MTT analysis (Figure 4c). Neither the Mo_2_C QDs nor the NIR laser irradiation exhibited cytotoxicity to the B16-10F cells. By contrast, almost all the cells were killed when the cells were incubated with Mo_2_C QDs plus an 808 nm laser treatment (0.64 W/cm^2^). The excellent PTT efficiency of the Mo_2_C QDs is probably due to the high photothermal conversion efficiency and good transfection efficiency. 

**Figure 5:**
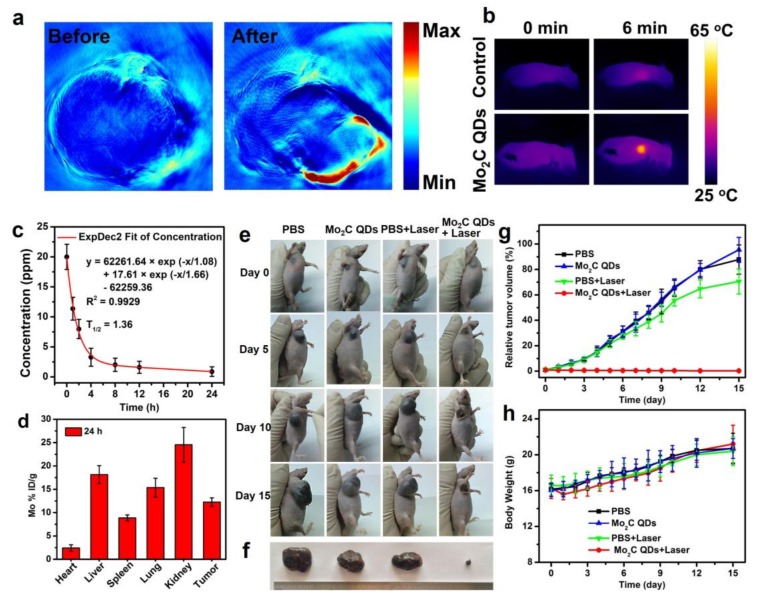
(**a**) In vivo PA images of a tumor-bearing mouse taken at before and after intravenous injection of Mo_2_C QDs; (**b**) IR thermal images at tumor site of a tumor-bearing mouse under an 808 nm laser irradiation (0.64 W/cm^2^) (**c**) The blood circulation lifetime of Mo_2_C QDs after intravenous injection into nude mice (*n* = 5); (**d**) The biodistribution of Mo in main tissues and tumor after intravenous injection for 24 h; (**e**) Photographs of tumor-bearing mice at different time points; (**f**) Photographs of tumors after the treatments; (**g**) The tumors growth curves and (**h**) body weight curves of tumor-bearing mice in different groups.

We further carried out in vivo experiments to test the feasibility of Mo_2_C QDs as theranostic nanoagents. Figure 5a shows representative PA images of a nude mouse bearing B16-10F tumor, which were acquired before and after intravenous administration of Mo_2_C QDs. As expected, negligible PA signal was detected at the tumor location before Mo_2_C QDs injection, while a strong PA signal was observed after injection, indicating the efficient tumor accumulation of Mo_2_C QDs through the enhanced permeability and retention (EPR) effect [41,42]. The local temperature change at tumor site location was also monitored (Figure 5b). After an 808 nm laser irradiation (0.64 W/cm^2^, 10 min), the tumor site temperatures of Mo_2_C QDs-treated mice increased from 27.9 to 63 °C, while the PBS-treated mice exhibited only a slight temperature increase.

The pharmacokinetics profile and biodistribution of the Mo_2_C QDs was measured to evaluate their blood circulation time and metabolism. As shown in Figure 5c, the circulation of the Mo_2_C QDs in bloodstream conformed well to the two-compartment model with a blood circulation half-time of 1.36 h. The quantification analysis shown in Figure 5d indicated high tumor accumulation of the Mo_2_C QDs (12.26% ID g^−1^) through the EPR effect. The significant accumulation in major organs, including liver, spleen, and kidney, were also observed. The high level of Mo_2_C QDs in liver and spleen was possibly owing to mononuclear phagocytic system absorption, and the accumulation in kidney could be correlated with renal excretion.

The in vivo PTT effect of Mo_2_C QDs were performed on B16-10F tumor-bearing nude mice, which were divided into four groups (*n* = 5): PBS injection; Mo_2_C QDs injection; PBS injection and 808 nm laser irradiation; and Mo_2_C QDs injection and 808 nm laser irradiation. The tumor volume and body weights of all these four group were measured as the function of time for 15 days (Figure 5e–h). Similar to PBS control group, neither Mo_2_C QDs without irradiation nor PBS with irradiation treatment showed antitumor capability. By contrast, the group of Mo_2_C QDs injection and 808 nm laser irradiation exhibited remarkable tumor growth inhabitation, which revealed the outstanding PTT efficacy of Mo_2_C QDs. Additionally, no obvious difference in the body weights were observed for all the mice, suggesting negligible systemic side effects of these treatments.

**Figure 6:**
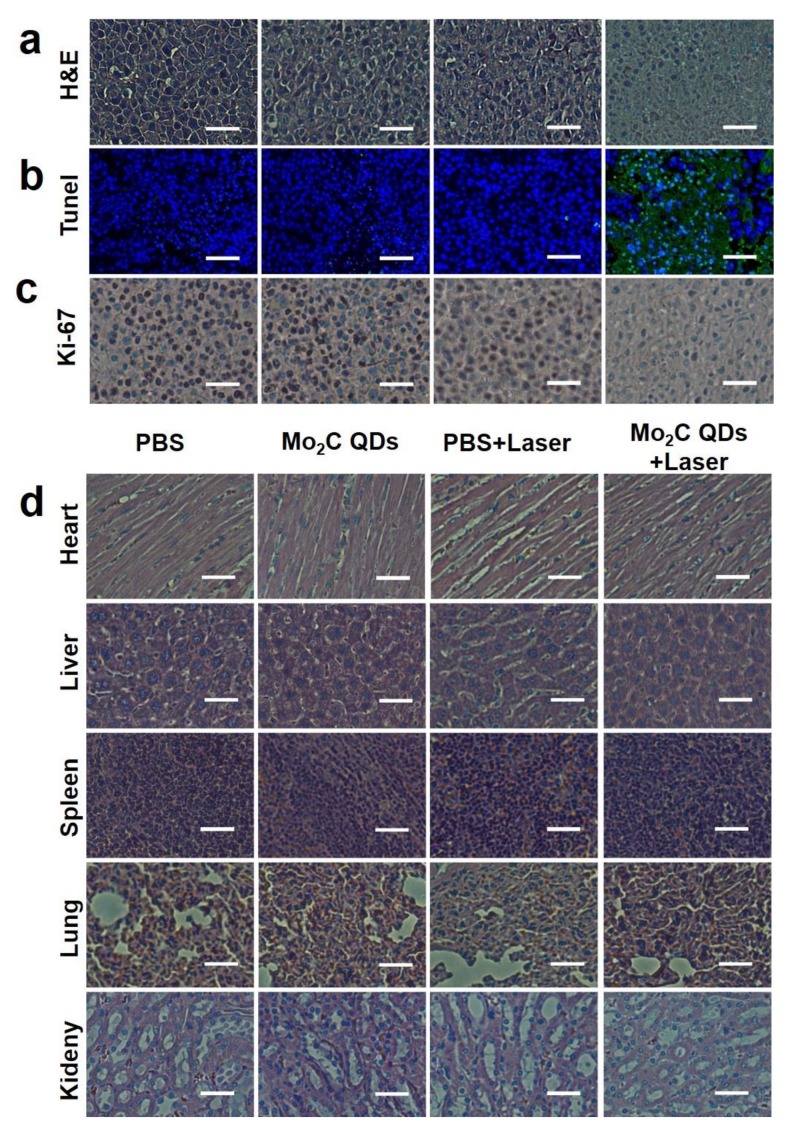
(**a**) Hematoxylin and eosin (H&E); (**b**) Antigen Ki-67 immunofluorescence and (**c**) terminal deoxynucleotidyl transferased UTP (Uridine triphosphate) nick end labeling (TUNEL) staining for pathological changes in tumor tissues from different groups after therapy; (**d**) H&E staining of major organs of each group. All the scale bars are 66 μm.

Hematoxylin and eosin (H&E) (Figure 6a) and terminal deoxynucleotidyl transferased UTP (Uridine triphosphate) nick end labeling (TUNEL) (Figure 6b) staining results clearly exhibited NO damage and apoptotic cells in mice that received Mo_2_C QDs injection combined with NIR laser irradiation compared to the groups treated with PBS, PBS + NIR laser, or Mo_2_C QDs alone. The in vivo proliferative activity of tumor cells, including the G2, M, and the latter half of the S phase, was analyzed by immunostaining against ki-67 [43,44]. Similar results were confirmed and are shown in Figure 6c. The potential in vivo toxicity or side effect is always a great concern for theranostic nanoagents used in medicine [43,45]. The H&E staining histology analysis of major organs showed no evident inflammations and adverse effect for Mo_2_C QDs-mediated PTT treatment (Figure 6d). In the mice receiving PBS injection only and the group receiving PBS injection combined with laser irradiation, nodular liver metastases were observed owing to the strong invasiveness of B16-10F melanoma cells. These results verified that Mo_2_C QDs could be a safe theranostic nanoagent serving in PA/PT imaging-guided PTT for personalized nanomedical applications.

## 4. Conclusions

For the first time, we have synthesized an all-in-one semimetal, ultrasmall Mo_2_C QDs nanoagent using a facile sonication-assisted liquid-phase exfoliation method for PA/photothermal imaging-guided PTT without extra-complicated modification. The Mo_2_C QDs displayed an advanced extinction coefficient, high photothermal conversion efficiency, and excellent photothermal stability as a result of the semimetal-like energy band structure and the related strong NIR LSPR properties. Low cytotoxicity, good biocompatibility and physiological stability were also revealed. The Mo_2_C QDs achieved sensitive dual-modal PA/PT tumor imaging diagnosis and highly efficient tumor ablation in vitro and in vivo with negligible adverse effect. The work has explored the biomedical application of Mo_2_C QDs for the first time and will contribute to the design of single theranostic nanoparticles with intrinsic imaging and therapeutic capabilities.

## Supplementary Materials

The following are available online at http://www.mdpi.com/1996-1944/11/9/1776/s1.
