# Supplementary material for: A Semimetal-Like Molybdenum Carbide Quantum Dots Photoacoustic Imaging and Photothermal Agent with High Photothermal Conversion Efficiency
Source: Materials (Basel). 2018 Sep 19;11(9):1776. doi: 10.3390/ma11091776 (PMC6163487; doi:10.3390/ma11091776)
Supplement: Supplementary file 1 [file materials-11-01776-s001.pdf]

A Semimetal-like Molybdenum Carbide Quantum Dots Photoacoustic Imaging and Photothermal Agent with High Photothermal Conversion Efficiency

Wenhao Dai [1,2], Haifeng Dong [1,2,*], and Xueji Zhang [1,2,*]

[1]Research Center for Biomedical and Health Science, Anhui Science and Technology University, Fengyang 233100, P.R. China.

[2] Research Center for Bioengineering and Sensing Technology, School of Chemistry and Bioengineering, University of Science & Technology Beijing, Beijing 100083, P.R. China.

*** Corresponding Authors:**

Prof. Haifeng Dong (E-mail: hfdong@ustb.edu.cn)

Prof. Xueji Zhang (E-mail: zhangxueji@ustb.edu.cn)

Experimental Section

Materials and regents. The molybdenum carbide (Mo_2C) powder and 3-(4,5-dimethyl-2-thiazolyl)-2,5-diphenyl-2-H- tetrazolium bromide (MTT) were purchased from Sigma-Aldrich (China). Dimethyl sulphoxide (DMSO) was obtained from Sinopharm Chemical Reagent Co., Ltd (Beijing, China). Hoechst 33342, calcein-AM and propidium iodide (PI) were obtained from Yeasen Biotech. Co., Ltd. (Shanghai, China). Phosphate buffer saline (PBS, pH 7.4), fetal bovine serum (FBS), Dulbecco's modified Eagle's medium (DMEM), trypsin-EDTA and penicillin-streptomycin were purchased from Gibco Life Technologies (AG, Switzerland). All other chemicals used in this study were analytical reagent grade and used without further purification. The ultrapure water was obtained from a Millipore water purification system (18 MΩ, Milli-Q, Millipore, USA).

Characterization of Mo_2C QDs. The morphologies of Mo_2C QDs were examined with a FEIF20 TEM (FEI, USA) and a JSM-6700FSEM) (JEOL, Japan. AFM measurement was carried out on NanoscopeIIIa (Digital Instrument, USA) under tapping mode. The UV-vis-NIR absorption was acquired with a UV-1800 spectrophotometer (Shimadzu, Japan) and processed with Origin Lab software. The confocal laser scanning microscopy (CLSM) images were acquired on a FV1200 microscope (Olympus). The quantitative analysis of elements was determined by inductively coupled plasma-optical emission spectrometry (ICP-OES, iCAP 7400, Thermo Fisher). The temperature was measured by a digital thermometer with a thermocouple probe and recorded once every 2 s. An infrared thermal imaging camera

(Fluke TiS65, USA) was used to monitor the temperature change.

Calculation of the extinction coefficient. To evaluate the NIR absorption capability of Mo_2C QDs, the extinction coefficient α of the Mo_2C QDs was calculated, according to the Lambert-Beer Law:

$$A=\alpha LC \tag{1}$$

where α represents the extinction coefficient, A represents the absorbance at the specific wavelength, L represents thickness of the absorption layer (1 cm), and C represents the concentration of the Mo_2C QDs. The extinction coefficient α is calculated by plotting the slope of each linear fit against wavelength. The extinction coefficient (α) at 808 nm laser of Mo_2C QDs can be measured to be 4.424 $Lg^{-1}cm^{-1}$.

Calculation of the photothermal conversion efficiency. Photothermal performance was measured using a custom setup. A 1 cm path length quartz cuvette containing 1 mL of the sample was covered with a variety of concentrations from 0-200 μg/mL. The bottom of the cuvette was kept about 0.5 cm above the magnetic stirrer. A fiber-coupled continuous semiconductor diode laser (808 nm, Hi-Tech Optoelectronics Co., Ltd, Beijing, China) with a power density of 0.64 W/cm^2 was used as the light source. The temperature of the solution was measured by a digital thermometer with a thermocouple probe and recorded once every 2 s. An infrared thermal imaging camera (Fluke TiS65, USA) was used to monitor the temperature change.

According to the method described in the literature,[1] the total energy conservation for the system can be expressed by equation 2.

$$\sum_{i} m_i C_{p,i} \frac{dT}{dt} = Q_{QDs} + Q_b - Q_{sur} \quad (2)$$

where m and C_p are the mass and heat capacity of solvent (water), respectively and T is the solution temperature. Q_{QDs} is the photothermal energy input from the Mo_2C QDs, which expresses heat dissipated by electronphonon relaxation of the plasmon on the Mo_2C QDs surface under the irradiation of 808 nm laser:

$$Q_{QDs} = I\,(1 - 10^{-A_{808}})\eta \quad (3)$$

where I is incident energy of laser power (mW), A_{808} is the absorbance of the Mo_2C QDs at 808 nm,

and η is the photothermal transduction efficiency. Besides, Q_b is the heat dissipated from light absorbed by the quartz cuvette sample cell, which was measured independently to be and found to be 73 mW using a quartz cuvette containing pure water without Mo_2C QDs. Moreover, Q_{sur} is in linear with temperature for the outgoing thermal energy, as the following equation 4:

$$Q_{sur} = hS\,(T - T_{sur}) \quad (4)$$

where h is heat transfer coefficient, S is the surface area of the container, and T_{sur} is ambient surrounding temperature. The lumped quantity hS was determined by measuring the rate of temperature drop after removing the light source.

Once the laser power is defined, the heat input (Q_{QDs}+ Q_b) will be finite. Because the heat output (Q_{sur}) is increased along with the rise of temperature according to the equation 4, the system temperature will reach a maximum when the heat input is equal to heat output:

$$Q_{QDs} + Q_b = hS\,(T_{max} - T_{sur}) \quad (5)$$

where T_{max} is the equilibrium temperature. The photothermal conversion efficiency (η) can be determined by substituting equation 3 for Q_{QDs} into equation 5 and rearranging to get

$$\eta = \frac{hS\,(T_{max} - T_{sur}) - Q_b}{I\,(1 - 10^{-A_{808}})} \tag{6}$$

where Q_b was measured independently to be 73 mW. Thus, only the hS remains unknown for the calculation of η. Here hS is calculated by introducing θ, which is defined as the expression below:

$$\theta = \frac{T - T_{sur}}{T_{max} - T_{sur}} \tag{7}$$

and a sample system time constant τ_s

$$\tau_s = \frac{\sum_i m_i\, C_{p,i}}{hS} \tag{8}$$

which is substituted into equation 5 and rearranged to yield

$$\frac{d\theta}{dt} = \frac{1}{\tau_s}\left[\frac{Q_{QDs} + Q_b}{hS\,(T_{max} - T_{sur})} - \theta\right] \tag{9}$$

At the cooling stage of Mo_2C QDs aqueous dispersion, the light source was shut off and $Q_{QDs} + Q_b = 0$, reducing equation 9 to

$$dt = -\tau_s \frac{d\theta}{\theta} \tag{10}$$

and integrating, giving the expression:

$$t = -\tau_s \ln\theta \tag{11}$$

Therefore, the time constant for heat transfer from the system was determined to be τ_s = 185.99 s for 808 nm by applying the linear time data from the cooling period (inset Figure 3E). In addition, m is 1 g and C is 4.2 J g^{-1}. Therefore, according to equation 8, hS can be determined. Substituting hS into equation 6, the 808 nm laser photothermal

conversion efficiency (η) of Mo_2C QDs can be calculated to be 42.9%.

Cell culture. The B16-F10 cells were cultured in Dulbecco's modified Eagle's medium (DMEM, GIBCO) supplemented with 10% fetal calf serum, penicillin (100 mg/mL), and streptomycin (100 mg/mL) at 37 ℃ in a humidified atmosphere containing 5% CO_2.

***In vitro* cytotoxicity assay.** B16-F10 cells (5.0×10^4) were cultured for 12 h in a 96-well plate containing DMEM (100 μL) in each well, and then the medium was replaced with fresh serum-free medium (Opti-MEM) alone or medium containing Mo_2C QDs and incubated for another 4 h. Next, MTT (20 μL, 5 mg/mL) with fresh DMEM (100 μL) was then added to each well. The media was removed 4 h later, and DMSO (100 μL) was added to solubilize the formazan dye. After shocking (37 °C, 120 rpm) for 15 min, the absorbance of each well was measured using a Tecan Sunrise at 488 nm. The cytotoxicity of Mo_2C QDs was estimated by the percentage of growth inhibition calculated with the formula.

Growth inhibition % = $(1 - A_{text}/A_{control})$ 100%

Pharmacokinetics and biodistribution. For pharmacokinetic study, B16-F10-tumor-bearing nude mouse were intravenously injected with Mo_2C QDs solution in PBS (n= 5). Blood samples were collected at varied time intervals (0.25, 1, 2, 4, 8, 12, and 24 h) after injection. The *in vivo* blood terminal half-life of Mo_2C QDs was calculated by a double-component pharmacokinetic model. The biodistribution of Mo_2C QDs in major organs and tumor was evaluated in B16-F10-tumor-bearing mouse (n=5). The tumor-bearing nude mouse were intravenously injected with Mo_2C QDs in

PBS. The major organs and tumor were digested by the similar method applied to the blood samples at 24 h. The collected samples were digested with the mixture of HNO_3/H_2O_2 (2:1 v/v) for quantification of Mo by ICP-OES. The *in vivo* blood terminal half-life of Mo_2C QDs was calculated by a double-component pharmacokinetic model.

Reference

1. Roper, D.K.; Ahn, W.; Hoepfner, M. Microscale heat transfer transduced by surface plasmon resonant gold nanoparticles. *J. Phys. Chem. C* **2007**, *111*, 3636-3641.
